# Immobilized Lipases on Functionalized Silica Particles as Potential Biocatalysts for the Synthesis of Fructose Oleate in an Organic Solvent/Water System

**DOI:** 10.3390/molecules22020212

**Published:** 2017-01-30

**Authors:** Vinicius Vescovi, Raquel L. C. Giordano, Adriano A. Mendes, Paulo W. Tardioli

**Affiliations:** 1 Postgraduate Program in Chemical Engineering, Department of Chemical Engineering, Federal University of São Carlos, 13565-905 São Carlos, SP, Brazil; viniciusv003@yahoo.com.br (V.V.); raquel@ufscar.br (R.L.C.G.); pwtardioli@ufscar.br (P.W.T.); 2 Institute of Chemistry, Federal University of Alfenas, 37130-001 Alfenas, MG, Brazil; adriano.mendes@unifal-mg.edu.br

**Keywords:** microbial lipases, immobilization, functionalized silica particles, fructose oleate synthesis, organic solvent/water system

## Abstract

Lipases from *Thermomyces lanuginosus* (TLL) and *Pseudomonas fluorescens* (PFL) were immobilized on functionalized silica particles aiming their use in the synthesis of fructose oleate in a *tert*-butyl alcohol/water system. Silica particles were chemically modified with octyl (OS), octyl plus glutaraldehyde (OSGlu), octyl plus glyoxyl (OSGlx), and octyl plus epoxy groups (OSEpx). PFL was hyperactivated on all functionalized supports (more than 100% recovered activity) using low protein loading (1 mg/g), however, for TLL, this phenomenon was observed only using octyl-silica (OS). All prepared biocatalysts exhibited high stability by incubating in *tert-*butyl alcohol (half-lives around 50 h at 65 °C). The biocatalysts prepared using OS and OSGlu as supports showed excellent performance in the synthesis of fructose oleate. High ester synthesis was observed when a small amount of water (1%, *v*/*v*) was added to the organic phase, allowing an ester productivity until five times (0.88–0.96 g/L.h) higher than in the absence of water (0.18–0.34 g/L.h) under fixed enzyme concentration (0.51 IU/g of solvent). Maximum ester productivity (16.1–18.1 g/L.h) was achieved for 30 min of reaction catalyzed by immobilized lipases on OS and OSGlu at 8.4 IU/mL of solvent. Operational stability tests showed satisfactory stability after four consecutive cycles of reaction.

## 1. Introduction

Lipases are extremely versatile enzymes widely used in hydrolysis, esterification, and transesterification reactions. However, the use of these enzymes in their soluble or aggregated forms is currently economically unattractive because the costs of these biocatalysts are still very high. Moreover, their operational stability is usually very low, particularly when they are expected to be used in an unusual medium, such as organic media. Several drawbacks of the soluble enzymes can be overcome by immobilization on solid supports, membranes, crosslinking, etc. [1,2,3,4,5,6]. Several protocols has been used to immobilize lipases, however the physical adsorption on hydrophobic supports has been the most applied because it is a simple, gentle, and cheap protocol of preparation of active and robust biocatalysts [7,8]. Lipases may behave atypically, yielding biocatalysts immobilized on hydrophobic supports widely stable (particularly in organic solvents where leaching is prevented) and hyperactivated (recovered activity greater than 100%) due to their atypical catalytic mechanism (interfacial activation), where a lid that covers the active site in most lipases is modulated from a closed form (inactive enzyme) to an open form (active enzyme) in presence of hydrophobic interfaces [8,9,10]. 

The hyperactivation phenomenon for lipases adsorbed on hydrophobic supports has been well-documented [2,4,7,11,12,13]. However, due to the labile forces involved in physical adsorption of proteins on solid surfaces, such as van der Waals forces and hydrogen linkages, lipases can be easily leached from the support surface, depending on reaction conditions [14,15]. Heterofunctional supports, i.e., supports chemically modified with different moieties to allow the coupling of the lipase by ionic or hydrophobic adsorption, followed by strong and irreversible covalent links between the adsorbed lipase and the activated support can be an interesting alternative to the supports conventionally used [16,17,18,19,20]. The application of heterofunctional supports aiming the stabilization and modulation of the catalytic properties of several monomeric or multimeric enzymes, including lipases, has been well-documented [20,21,22,23,24,25]. The immobilization of enzymes on these supports occurs via physical adsorption under mild reaction conditions (neutral pH and low ionic strength) by different interactions, including ionic exchange, hydrophobic and hydrogen linkages, followed by covalent attachment of nucleophilic groups from the enzyme (hydroxyl, amino, thiol, etc.) with electrophilic groups from the support surface (epoxy and aldehyde groups) by incubating at alkaline condition (pH 10). This incubation at pH 10 is an important step to achieve a high interaction between enzyme and support because the reactivity of lysine residues (pKa around 10.5) may be improved, which promotes the rigidification of their three-dimensional structure [17,20,21]. Agarose beads have been the most used matrix in the preparation of heterofunctional supports for further immobilization of lipases [15,26,27,28,29,30,31]. These studies have focused in the modulation of their catalytic properties, including stability and selectivity towards several substrates in hydrolysis and esterification reactions, due to possible alteration of their microenvironments and different orientations of these enzymes on the support surface. The application of silica particles as support is highly interesting due to its higher surface area than agarose beads that allows the retention of high protein concentration and improves the accessibility of substrate molecules to the biocatalyst microenvironment [32,33]. However, the preparation of these functionalized supports by applying silica particles is still few reported in the literature. Some microbial lipases have been immobilized on heterofunctional based-silica supports to catalyze both hydrolysis and esterification reactions such as lipase B from *Candida antarctica* (CALB), *Geobacillus thermocatenulatus* (BTL2), *Pseudomonas stutzeri* (PsL), and *Alcaligenes* sp. (AsL) [19,20,34,35,36].

In this study, two microbial lipases (TLL and PFL) were immobilized on silica heterofunctionalized with octyl and epoxy or aldehyde groups aiming their application in the synthesis of an industrial important class of biosurfactants, i.e., sugar fatty esters. The immobilization of these lipase preparations on heterofunctional silica particles and their potential application as biocatalysts in sugar ester synthesis has not been reported yet. Sugar fatty esters are biodegradable non-ionic surfactants that can be synthesized from renewable and readily available sources [37]. They are used in the food industry as emulsifiers, foaming agents, viscosity reducers, etc [37]. Other fields of application include cosmetics, detergents, oral-care products, and medical supplies [38]. The synthesis of sugar fatty acid esters can be catalyzed by chemical and enzymatic catalysts. The enzymatic catalyst has many advantages, such as, eco-friendly reaction conditions and high substrate selectivity and product specificity [39]. 

The enzymatic synthesis of esters is typically performed in organic solvents with a low water activity in order to favor the esterification instead of hydrolysis reaction [40]. On the other hand, the presence of water may contribute significantly to solubilize high amounts of fructose, thus allowing a high concentration of sugar and thus high productivity. In this study, all biocatalysts prepared were evaluated in the synthesis of fructose oleate in *tert*-butyl alcohol in absence or presence of water (1%, 5%, and 10% *v*/*v*).

## 2. Results and Discussion

### 2.1. Immobilization of Lipases

**Figure 1:**
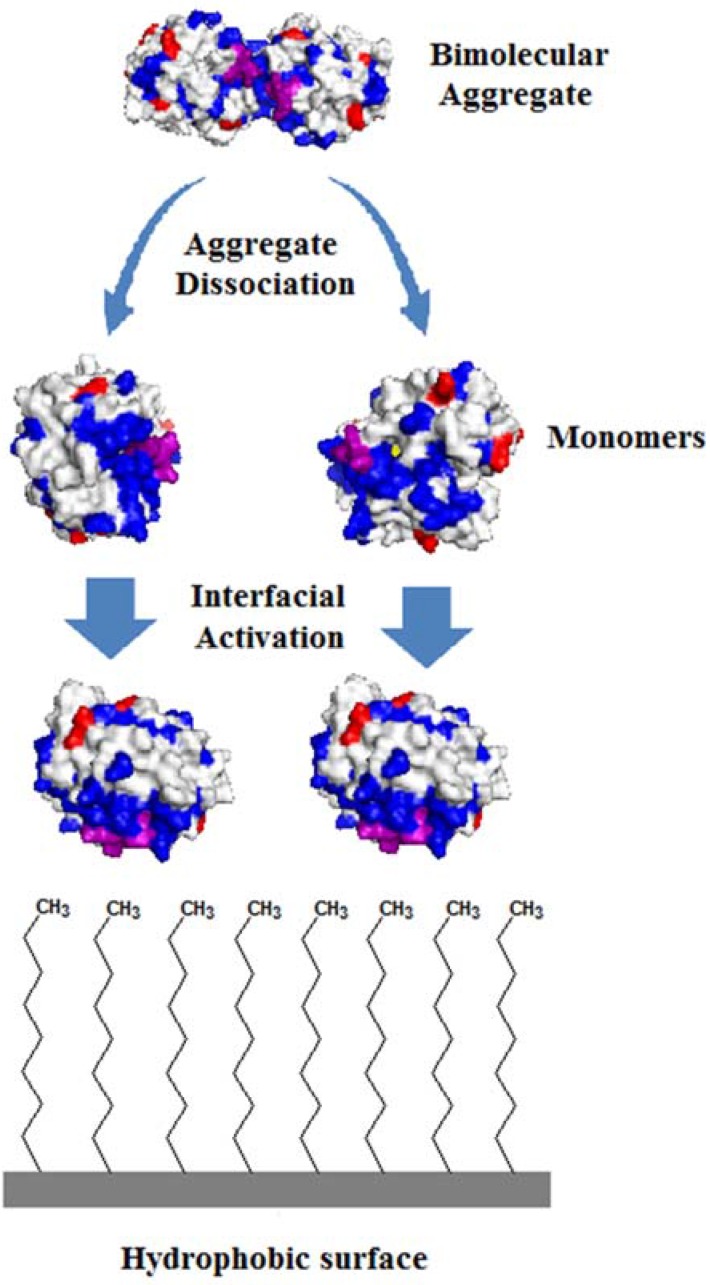
Schematic representation of dissociation of a TLL bimolecular aggregate in the presence of a hydrophobic surface (octyl-surface as an illustration). Bimolecular aggregate and monomer of TLL were obtained from Protein Databank (codes PDB 1DT3 and 1TIB, respectively). 3D-structures were constructed using PyMOL Molecular Graphics System, v. 1.7.4 (Schrödinger, LLC, Portland, OR, USA). Different colors represent the lid (purple), active site (yellow), lysine residues (red), and hydrophobic areas (blue).

Functionalized silica particles were evaluated in the immobilization of PFL and TLL. These biocatalysts were prepared by using initial protein loadings of 1 mg/g and 10 mg/g of support. The characterization of these supports was performed in previous studies by hydrophobicity tests (adsorption of a hydrophobic dye—Rose Bengal) [7,20], and elemental analysis (CNHS) [20]. These tests confirmed the increase of the hydrophobicity of the supports prepared in relation to non-functionalized silica particles, which is more appropriate for the immobilization and stabilization of lipases in open conformation due to their affinity towards hydrophobic surfaces. Moreover, high density of functional groups (octyl- or reactive groups—epoxy and aldehyde) was also observed by CNHS analysis which allows an intense interaction of enzyme molecules with the support surface, thus preventing possible desorption from the biocatalyst microenvironment. As can be observed in Table 1, high immobilization yield varying from 80.4% (PFL immobilized on OSEpx for 1 mg protein/g of support) to 96.9% (TLL immobilized on OS for 10 mg protein/g of support) was obtained, which shows the high affinity of these lipases for all functionalized silica-based supports. The immobilization of TLL and PFL on silica surface chemically modified with hydrophobic/hydrophilic moieties allowed hyperactivation of lipases (recovered activities above 100%) using the lowest initial protein loading—1 mg/g, a typical behavior reported for PFL and TLL immobilized on hydrophobic supports [2,4,7,10,13,41,42,43]. The hyperactivation of PFL and TLL immobilized on monofunctional support (OS) may be associated to dissociation of bimolecular aggregates (Figure 1). 

Soluble PFL and TLL exhibit high tendency to form bimolecular aggregates (less active and more stable) in solution [4,44,45], and in these aggregates the hydrophobic active sites of the monomers (more active and less stable) are in close contact with each other [4]. Thus, the immobilization of PFL and TLL on hydrophobic supports should have favored the dissociacion of these bimolecular aggregates. Particularly, PFL has practically no hydrophobic areas on its surface, except for the inner face of the lid and the area surrounding the active site. Therefore, any hydrophobic interaction between PFL and support should be related to these hydrophobic areas [3]. Indeed, Lima et al. [4] and Kopp et al. [13] reported the hyperactivation of immobilized PFL on hydrophobic octyl-Sepabeads (300% recovered activity), octyl-Sepharose (150% recovered activity), and silica magnetic microparticles functionalized with octyl groups (163% recovered activity). Hyperactivation of TLL immobilized on hydrophobic supports was also previously reported by Martinelle et al. [11].

TLL immobilized on OSGlu, OSEpx, and OSGlx did not exhibit hyperactivation, except for OS. These results suggest that the enzyme-enzyme interaction for TLL is very strong. In order to promote the dissociation of these bimolecular aggregates, the application of hydrophobic supports is required. This feature have been reported as more active PFL biocatalysts as more hydrophobic the support [7]. In this way, the lower hydrophobicity of the supports OSGlu, OSEpx, and OSGlx than OS [20], most likely allowed the immobilization of the lipase in its closed form (inactive form) and as bimolecular aggregates with active sites facing each other, once that, the TLL has sufficient hydrophobic residues to be absorbed without the dissociation of the bimolecular aggregates. On the other hand, the support OS must have occurred from an assorted immobilization of TLL, i.e., the enzyme was immobilized in aggregate and in monomer form due to the high hydrophobicity of this support.

It should also be taken into account that, at the end of immobilization reactions, the supports and the microenvironments of the immobilized lipases exhibits different moieties, as follows: hydrophobic (OS), hydrophobic/hydrophilic (OSGlx), and hydrophobic/negatively-charged (OSEpx). The hydrophilic moieties of the OSGlx are due to reduction of the remnant aldehyde groups to hydroxyl groups [46], and the negatively-charged moieties of the OSEpx are due to carboxylic groups inserted in the blocking of the epoxy groups with glycine [47]. The hydrolytic activities using *p*-NPB (a molecule containing a charged moiety) as substrate are performed at pH 7, and at this pH the carboxylic groups are predominantly deprotonated. These different moieties could strongly influence the partition of the *p-*NPB from the bulk reaction to closer of the immobilized enzyme, yielding lower or higher activities depending to the substrate concentration closer to the enzyme. This effect was proved through TLL immobilization on OSEpx with, and without, glycine. The use of glycine represented an increase around 8% and 25% in the recovery activity and hydrolytic activity (*p*-NPB), respectively (data not shown). According to Table 1, the phenomenon of hyperactivation was not observed for the biocatalyst prepared with initial protein loading of 10 mg/g of support. These results could be credited to the increase of immobilized protein concentration in the inner part of the particle, thus causing a lesser apparent initial activity, i.e., in the case of initial activity using easily enzyme-converting substrates diffusional delays are remarkably evident, because the substrate molecules are rapidly converted by the enzyme molecules nearer to the particle surface [23,24].

Regarding the hydrolytic activity, the most active biocatalysts were prepared with monofunctional support (OS) due to high dissociation of bimolecular aggregates towards the monomolecular form by using a highly hydrophobic support, as mentioned above. In this study, OS is the most hydrophobic support which was confirmed by adsorption of a hydrophobic dye (Rose Bengal) performed in a previous study [20]. In general, the hydrolytic activity of biocatalysts increases by increasing the protein loading [7,20,48]. Here, this increase of hydrolytic activity was observed for all the biocatalysts prepared by immobilizing TLL and PFL on OS and OSGlu. According to Table 1, the highest increment of hydrolytic activity by increasing the initial protein loading from 1 to 10 mg/g was verified for PFL immobilized on OS and OSGlu that varied from 20.3 to 132.7 IU/g (increase of around six times) and from 9.8 to 96.4 IU/g (increase of around 10 times), respectively. On the other hand, no increment of activity for PFL-OSEpx (from 13.4 to 12.6 IU/g) and PFL-OSGlx (from 13.3 to 14.6 IU/g) may be observed. These results show that the mass transport of *p*-NPB (a substrate containing a hydrophobic group) from the bulk reaction to the internal microenvironment of these biocatalysts was severely hampered not only by diffusional delays, but also by the hydrophilic or negatively-charged microenvironment of the biocatalysts PFL-OGlx and PFL-OEpx, respectively. At low protein loading, a good dispersion may have favored the immobilization of PFL preferentially as monomers, allowing easier diffusion of *p*-NPB molecules to the unblocked enzyme active sites, which could be confirmed by RA values (hyperactivation of this enzyme). On the other hand, the preparation of these biocatalysts using high protein loading could have favored the immobilization of PFL as bimolecular aggregates, hindering the access of the *p*-NPB to the enzyme’s active site. These results clearly confirm the different mechanism of immobilization of both lipases on the support surfaces due to different hydrophobic/hydrophilic moieties obtained for each functionalization protocol. This is an important strategy in the preparation of versatile and active biocatalysts for further application in the synthesis of compounds of industrial interest as sugar esters.

### 2.2. Stability of Immobilized Lipases Against tert-Butyl Alcohol

**Figure 2:**
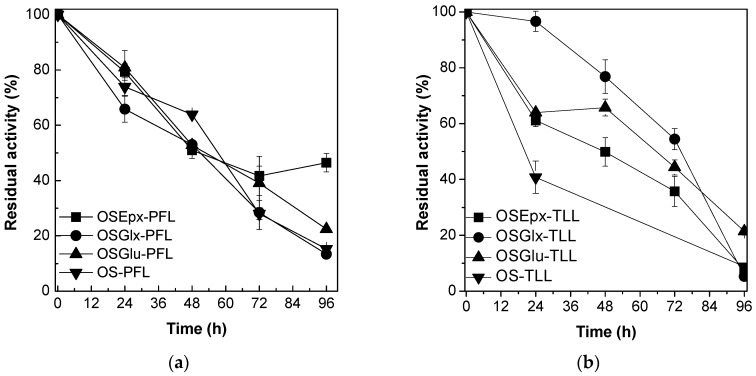
Residual activities of immobilized PFL (**a**) and TLL (**b**) on silica-based supports at 65 °C incubated in *tert*-butyl alcohol.

The stability of the biocatalysts previously prepared at low initial protein loading was performed in *tert*-butyl alcohol, a hydrophilic solvent widely used as reaction medium in the enzymatic synthesis of sugar esters [40]. In this set of experiments, the biocatalysts were incubated at 65 °C in order to allow the determination of all half-lives in a short period of incubation [20]. Figure 2 shows that PFL and TLL immobilized on mono and heterofunctional silica-based supports exhibit high stability in *tert*-butyl alcohol at high temperature (half-lives above 50 h at 65 °C). The high stabilities of the biocatalysts make them excellent features for industrial purposes, like in the process of synthesis of fructose oleate, where *tert*-butyl alcohol is required as reaction medium due to higher solubility of fructose in this solvent compared to other organic solvents.

### 2.3. Solubility of Fructose in tert-Butyl Alcohol/Water System

Sugars are hardly solubilized in organic solvents, such as *tert*-butyl alcohol [49,50]. The addition of small amounts of water could to increase the fructose solubility in the reaction medium and to prevent the enzyme inactivation. Indeed, the solubility of fructose in water/*tert*-butyl alcohol system was greatly increased (more than 4 times) from 0% to 10% *v*/*v*, as shown in Table 2. The high solubility of fructose is attractive under an industrial point of view because high concentration of solubilized fructose allows increasing the productivity of sugar ester. Thus, further tests were then performed in *tert*-butyl alcohol/water systems at different water content and their performances were compared with the system without addition of water.

### 2.4. Synthesis of Fructose Oleate in tert-Butyl Alcohol/Water System 

**Figure 3:**
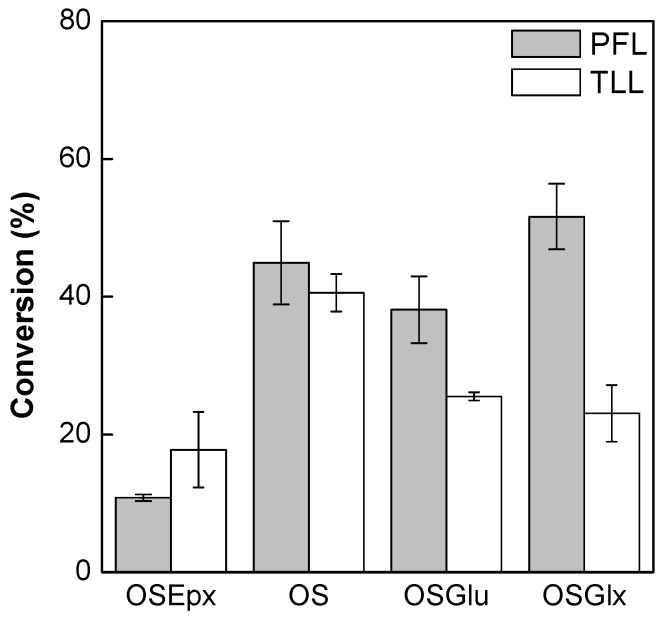
Synthesis of fructose oleate at 35 °C for 12 h of reaction using molar ratio fructose/oleic acid of 1:5 in *tert*-butyl alcohol medium (0.51 IU/mL of solvent) without the addition of water.

In this study, the synthesis of fructose oleate via esterification reaction was performed at fixed catalytic activity (0.51 IU/mL of solvent, based on the hydrolytic activity of the biocatalysts prepared with initial protein loading of 10 mg/g of support—Table 1) in order to select the most active biocatalysts for further tests. This enzymatic concentration was chosen based in a previous study performed in our lab for the enzymatic synthesis of ester catalyzed by immobilized CALB on these same supports [20]. Figure 3 shows the conversions of the esterification reactions catalyzed by PFL and TLL immobilized on mono and heterofunctional silica-based supports in a water-free medium. Low conversions (below 50%) were obtained with all biocatalysts and the lowest conversions were obtained with PFL and TLL immobilized on OSEpx. Probably, the microenvironment negatively charged of these biocatalysts may have hindered the mass transfer of the oleic acid to the active site of the lipases. Thus, these biocatalysts were discarded in subsequent studies. 

Water plays a very important role in the synthesis of sugar ester in organic medium. If, on the one hand, an accumulation of water produced by the reaction forces the reaction equilibrium towards hydrolysis, instead of towards ester synthesis [40,51,52], a certain amount of water is essential for hydration and stabilization of the three-dimensional structure of lipases [40,52], particularly in the presence of organic solvents, especially the polar solvents, which can penetrate into the active site of enzymes causing the unfolding of proteins to occur due to disturbances in these forces [53]. Moreover, the water content also influences the productivity because its presence in the reaction medium significantly increases the solubility of sugar in the reaction mixture. 

**Figure 4:**
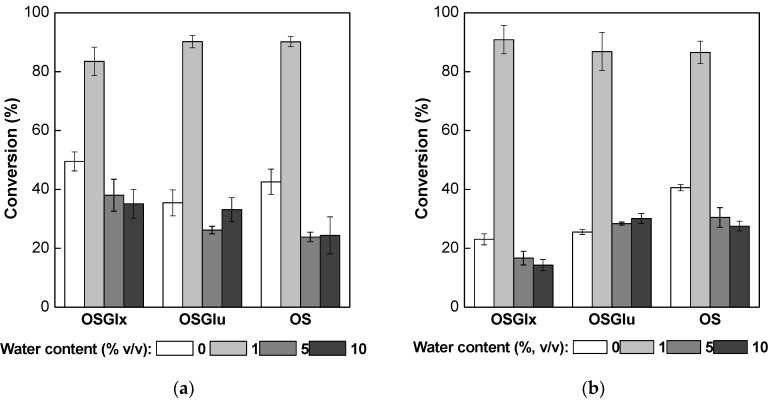
Synthesis of fructose oleate at 35 °C for 12 h of reaction using molar ratio fructose/oleic acid of 1:5 in *tert*-butyl alcohol/water systems (0.51 IU/mL of solvent) catalyzed by immobilized PFL (**a**) and TLL (**b**).

Figure 4a,b shows that a small amount of water (1%, *v*/*v*) in the reaction medium greatly increased the conversions of oleic acid to fructose oleate. All biocatalysts exhibited conversion of around 90% for 12 h of reaction while, in the absence of water, all conversions were below 50%, as described above (Figure 3). Although the solubility of fructose was favored by high water concentration, the formation of ester was hindered probably due to the shifting the thermodynamic equilibrium towards the hydrolysis reaction. Indeed, Leitgeb and Knez [54] also report a significant increase in the conversion of the synthesis of butyl oleate catalyzed by Mucor miehei lipase in the presence of small amounts of water. The improvement in the conversions was attributed to the better performance of the enzyme due to hydration of its three-dimensional structure. On the other hand, the presence of large amount of water displaced the equilibrium towards hydrolysis reaction.

According to results in Figure 4a,b, the presence of water caused significant change in the productivity of the ester. The productivity of fructose oleate in the presence of water was around 2–5 times higher than in the water absence, as shown in Table 3. However, the increase of water concentration in the reaction mixture reduced the conversion due to the shifting the thermodynamic equilibrium towards the hydrolysis reaction, though the productivity was dramatically increased. This increase is explained by the higher concentration of fructose in the reaction mixture compared to the system without the addition of water, as previously reported in Table 2. This different approach could reduce the use of organic solvent and, at the same time, allows a higher productivity of ester. 

**Figure 5:**
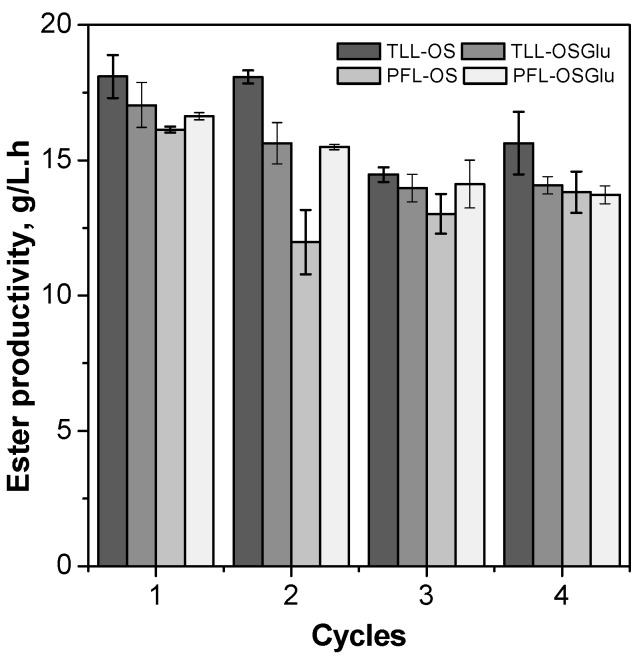
Operational stability of TLL-OS, PFL-OS, TLL-OSGlu and PFL-OSGlu in the synthesis of fructose oleate at 35 °C in *tert-*butyl alcohol containing 1% *v*/*v* of water, using a fructose/oleic acid molar ratio of 1:5, 8.4 IU/mL solvent (*p*-NPB activity) for 30 min of reaction.

Although high values of productivity have been obtained for the reaction mixtures with excess of water added, especially 10% *v*/*v*, the system with 1% *v*/*v* of water was chosen for subsequent studies because the conversion was significantly higher than other systems, thus providing a loss of substrate at the very bottom. Aiming the increase of the ester productivity, a new synthesis of fructose oleate was performed. For this purpose, the supports OS and OSGlu were chosen due their good performance (high catalytic activity and stability in *tert*-butyl alcohol). In this new synthesis, the experimental conditions were: fructose/oleic acid molar ratio of 1:5, temperature of 35 °C, amount of water (1%, *v*/*v*) and enzymatic loading of 8.4 IU/mL of solvent. The increase of enzymatic loading from 0.51 to 8.4 IU/mL of solvent allowed ester productivity up to 18.1 g/L.h (first batch using TLL-OS, as shown in Figure 5), resulting in conversion around 70% of the ester in only 30 min reaction (from 30 min to 8 h was not observed significant increase in the ester conversion (data not shown), therefore, greatly decreasing the ester productivity). These results are very promising compared to ones reported in previous studies in the literature. Cui et al. [55] reported a productivity of 8.67 g/L.h in the best conditions to d-isoascorbyl palmitate synthesis catalyzed by Novozym 435. Ferrer et al. [56] reported a productivity of 1.25 g/L.h in the best conditions to synthesis of the 6-*O*-lauroylsucrose in *tert*-butyl alcohol/dimethyl sulfoxide (DMSO) (1/4, *v*/*v*) catalyzed by TLL immobilized on celite. Reyes et al. [57] reported a productivity of 0.375 g/L.h to sucrose palmitate synthesis catalyzed by Novozym 435 in *tert*-butyl alcohol medium. The high productivity obtained in this study could be increased, through the use of a continuous reactor and increase of temperature, which can allow better sugar concentration in the reaction mixture. Indeed, Vescovi et al. [20] reported a high concentration of fructose (130 mM) solubilized in anhydrous *tert*-butyl alcohol at 60 °C.

### 2.5. Operational Stability Tests

The reusability of immobilized enzymes is very important for their application, especially on an industrial scale [48]. Thus, in this study the biocatalysts PFL-OS, TLL-OS, PFL-OSGlu and PFL-OSGlu were employed in operational stability study using the better experimental conditions established in a previous study [20]: fructose/oleic acid molar ratio of 1:5, enzymatic loading of 8.4 IU/mL of solvent, 1% *v*/*v* of water, 35 °C, and 30 min of reaction. 

These biocatalysts showed high operational stability in the synthesis of fructose oleate (Figure 5). The high stability of these immobilized biocatalysts towards *tert*-butyl alcohol enabled their reuse in four batches, with a small decrease of ester productivities (16.1–18.1 g/L.h for the first batch, slightly dropping to 13.7–15.6 g/L.h for the fourth batch). These results suggest strong interaction of both lipases with the support microenvironment. The slight decrease observed in ester productivity could be due to possible accumulation of water molecules in the internal microenvironment of the support and/or inactivation or desorption of some enzyme molecules preferentially immobilized via physical adsorption on the support surfaces.

### 2.6. Literature Survey for the Enzymatic Synthesis of Fructose Oleate

The application of immobilized lipases in the synthesis of fructose oleate has been well-documented [58,59,60,61,62]. These studies report the preferential application of immobilized lipases commercially available from Novozymes, such as Lipozyme (immobilized lipase from Mucor miehei on a macroporous anion-exchange resin) and Novozym 435 (immobilized lipase B from Candida antarctica on a macroporous acrylic resin). In a pioneering study published in 1991, Khaled et al. [63] produced this ester via an esterification reaction performed in a packed bed reactor (PBR) using lipozyme as biocatalyst. The reaction was performed in 2-methyl-2-butanol medium by using fructose:oleic acid at a molar ratio of 1:10 and a reaction temperature of 55 °C. According to these authors, the steady rate was reached for 6 h and maximum conversion was around 50%. Coulon et al. [58] produced fructose oleate by esterification (fructose + oleic acid) and transesterification (fructose + methyl oleate) reactions in a batch system. The experimental conditions were as follows: fructose:oleic acid at a molar ratio of 1:5 (1.11 mmol:5.55 mmol) and at 60 °C in 200 mL of 2-methyl-2-butanol. Under these conditions, maximum conversions of 65% and 46% were obtained after 10 h of reaction via transesterification and esterification reactions, respectively. In a subsequent study, the influence of several factors on the ester synthesis via transesterification reaction in 2-methyl-2-butanol medium catalyzed by Novozym 435 was evaluated [59]. Under optimal conditions (5 g/L of biocatalyst, 100 rpm, and fructose:methyl oleate at a molar ratio of 1:5), high concentration of fructose monooleate (50 g/L) was reached at 90 °C, with an initial rate of 90 g/L.h, after 10 h of reaction. After six successive cycles of reaction at 60 °C, a drastic decrease of activity was observed. CALB was also used as the biocatalyst in the synthesis of fructose oleate using 2-methyl-2-butanol as solvent and maximum ester yield (around 70%) was obtained for 24 h of reaction [60]. Patil et al. [61] produced this ester via esterification and transesterification reactions in 2-methyl-2-butanol medium catalyzed by Lipase B from *Candida antarctica* by using an initial fructose concentration of 25 mM. The authors observed maximum ester yield of 21% (transesterification reaction) for 12 h of reaction at 65 °C. In a recent study, the ester was synthetized in a solvent-free system using Novozym 435 as biocatalyst [62]. The esterification reaction was conducted at 65 °C and four days of incubation, which yielded an ester conversion of 96%. Based on these results, the biocatalysts prepared in this study by immobilizing TLL and PFL on mono- (OS) and heterofunctional (OSGlu) silica particles required milder temperature (35 °C) and shorter reaction time (30 min) to yield high fructose oleate concentration compared to the previous studies reported above. These studies focused the production of the ester, however operational stability tests after consecutive cycles of the reaction in a batch system is rarely reported, as can be observed. The different biocatalysts prepared in this study were *satisfactorily* stabilized after four cycles of reaction, thus indicating possible stabilization of both microbial lipases by using silica-based supports (OS and OSGlu). This confirms the potential application of these biocatalysts in the production of an important ester from the industrial point of view of fructose oleate. 

## 3. Experimental Section

### 3.1. Materials

(3-Glycidyloxypropyl)trimethoxysilane (GPTMS), triethoxy(octyl)silane (OCTES), (3-aminopropyl)triethoxysilane (APTES), Lipolase 100L (lipase from *Thermomyces lanuginosus*, TLL, ≥100,000 IU/g) and Amano lipase from *Pseudomonas fluorescens* (PFL, ≥20,000 IU/g), bovine serum albumin, and *p*-nitrophenyl butyrate (*p*-NPB) were purchased from Sigma-Aldrich Co. (St. Louis, MO, USA). *tert-*Butyl alcohol and glutaraldehyde solution (25% *v*/*v*) were purchased from Vetec Química Fina Ltda (Duque de Caxias, RJ, Brazil). Fructose, sodium metaperiodate, sodium borohydride, and oleic acid were purchased from Synth (Diadema, SP, Brazil). Silica particles (Immobead S60S) were purchased from Chiral Vision (Leiden, The Netherlands). All other chemicals were of analytical grade from Synth and Vetec Química Fina Ltda (São Paulo, Brazil).

### 3.2. Methodology

In this study, all the results were expressed as mean of triplicate ± standard deviation.

#### 3.2.1. Support Activation

Silica particles (pore diameter 193 Å) were chemically modified according to the methodology described by Vescovi et al. [20]. Prior to activation, silica was treated with HCl solution (0.1 M) under reflux, followed by washing with distilled water until neutral pH and drying at 50 °C for 12 h.

1. Octyl-silica (OS): 1 g of dry silica and 20 mL of OCTES:toluene solution (1:10, *v*/*v*) was kept under reflux for 4 h at the boiling temperature of the solvent. OS was washed with toluene and distilled water, and dried at 40 °C for 24 h.

2. Octyl-silica-amino-glutaraldehyde (OSGlu): 1 g of dry silica was suspended in 1.5 mL of OCTES and 0.5 mL of APTES (dissolved in 18 mL of toluene), kept at solvent boiling temperature under reflux for 4 h, washed with toluene, and dried at 40 °C for 24 h. The support activation with aldehyde groups was performed immediately before its use in the lipase immobilization by suspending 1 g of dry silica modified with octyl and amino groups (OS-amino) in 10 mL of 0.2% (*v*/*v*) glutaraldehyde solution prepared in buffer sodium phosphate (100 mM, pH 7.0). The suspension was stirred for 1 h at room temperature. The activated support was then recovered by filtration and washed with an excess of distilled water.

3. Octyl-silica-epoxy (OSEpx): 1 g of dry silica was suspended in 1.5 mL of OCTES and 0.5 mL of GPTMS (dissolved in 18 mL of toluene), kept at solvent boiling temperature under reflux for 4 h, washed with toluene and distilled water, then dried at 40 °C for 24 h.

4. Octyl-silica-glyoxyl (OSGlx): 1 g of OSEpx was suspended in 30 mL of 0.1 M sulfuric acid solution and kept under reflux at 85 °C for 2 h. The support modified with octyl and glyceryl groups was washed with toluene and distilled water, dried at 40 °C for 24 h, and suspended in 0.1 M sodium metaperiodate solution (1:10 mass/volume ratio). After 1 h stirring in a GyroTwister (GX-1000 3-D Shaker from Labnet International, Inc., Edison, NJ, USA) at room temperature, the activated support was washed with distilled water and dried at 40 °C for 24 h.

#### 3.2.2. Immobilization of TLL and PFL

The immobilization of the lipases TLL and PFL on mono and heterofunctionalized silica was performed according to the methodology described by Vescovi et al. [20]. For the immobilization on OS and OSGlu, 1 g of support was suspended in 15 mL of lipase solution prepared in 10 mM sodium phosphate buffer (pH 7.0) containing 1 or 10 mg of protein. After 6 h stirring in a GyroTwister Shaker at room temperature, the biocatalysts were recovered by filtration, washed with distilled water, dried by vacuum suction, and stored at 4 °C for further use. For the immobilization on OSEpx and OSGlx, first 1 g of support was suspended in 15 mL of lipase solution prepared in 10 mM sodium phosphate buffer (pH 7.0) containing 1 or 10 mg of protein (hydrophobic adsorption step). After 6 h stirring in a GyroTwister Shaker at room temperature, the biocatalysts were recovered by filtration, resuspended in 15 mL of 100 mM sodium carbonate buffer (pH 10.0), and stirred for 3 h (covalent linkage step). Afterwards, the biocatalysts prepared on OSGlx and OSEpx were treated with 1 mg/mL sodium borohydride and 3 M glycine solutions, respectively, for 1 h of incubation at room temperature. The immobilization yield was monitored by measuring the protein concentration by Bradford’s method [64], and by determining the *p*-NPB hydrolytic activity in the immobilization supernatants. The recovered activity percentage was calculated after determining the activity of the biocatalysts (apparent hydrolytic activity) and comparing with the number of activity units that disappeared from the supernatant (theoretically immobilized). All biocatalysts were loaded with 1 mg of protein/g of support to prevent diffusional delays, which could lead to wrong understanding of the results of recovered activity and thermal stability in organic solvent. On the other hand, the production of fructose oleate by esterification reaction was performed by using the biocatalysts prepared with initial protein loading of 10 mg/g of support aiming to increase the ester productivity.

#### 3.2.3. *p*-NPB Activity Assay

The hydrolysis activity of soluble TLL and PFL was assayed in a glass cuvette, measuring the absorbance increase at 405 nm caused by the release of *p*-nitrophenol (molar extinction coefficient of 6080 M^−1^·cm^−1^) during then hydrolysis of *p*-NPB solution (25 µL of *p*-NPB dissolved in 50 mM acetonitrile and 2 mL of 25 mM sodium phosphate buffer at pH 7.0) at 25 °C [11]. One international unit of hydrolysis activity (IU) was defined as the initial rate of hydrolysis of *p*-NPB (µmol/min) under the conditions described above. The hydrolytic activity of the immobilized TLL and PFL was determined using a mechanically stirred 25 mL reactor fed with 0.25 mL of *p*-NPB solution prepared in 50 mM acetonitrile and 20 mL of 25 mM sodium phosphate buffer at pH 7.0. Samples were withdrawn in 1 min intervals for quantification at 405 nm of the *p*-nitrophenol released [20].

#### 3.2.4. Thermal Stability in *tert*-Butyl Alcohol

The stabilities of all biocatalysts prepared in this study were evaluated by incubating them in pure *tert*-butyl alcohol at 65 °C. Samples were periodically withdrawn for measurement of the residual *p*-NPB hydrolysis activity. A blank in the same conditions (without enzyme) was used as control to verify any interference by *tert*-butyl alcohol in the hydrolysis of *p*-NPB.

#### 3.2.5. Solubility of Fructose

The solubility of fructose in *tert*-butyl alcohol (anhydrous or containing small amounts of water, e.g., 1, 5, and 10%, *v*/*v*) was evaluated by incubating 2.5 M fructose solutions in closed tubes at 35 °C for 24 h under stirring in a Marconi incubator (MA 430/1) equipped with 360°- stirring carrousel (Marconi, Piracicaba, SP, Brazil). After, the solutions were centrifuged at 10,000 rpm (12,857× *g*) and 35 °C by 2 min. Samples (1 mL) of the supernatant were dried in an oven at 70 °C for 24 h. The dry extract was then solubilized in 1 mL of water and the fructose concentration was determined in a Water high performance liquid chromatograph equipped with a refractive index detector and a Sugar Pak-I column (300 mm × 6.5 mm × 10 µm) kept at 80 °C. Ultrapure water was used as eluent at a flow rate of 0.5 mL/min.

#### 3.2.6. Synthesis of Fructose Oleate

**Figure 6:**
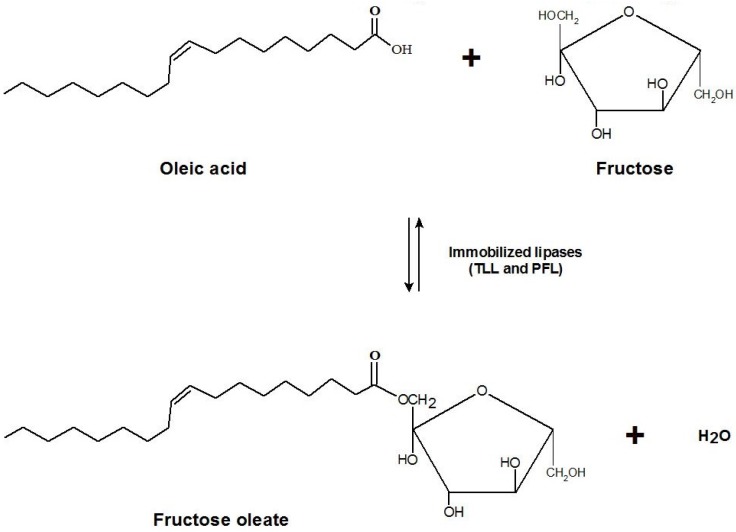
Schematic representation of the enzymatic synthesis of fructose oleate.

The reactions were performed at 35 °C in closed flasks containing 5 mL of fructose solutions prepared in *tert*-butyl alcohol (anhydrous or containing different amounts of water, e.g., 1%, 5%, and 10% *v*/*v*). All experiments were performed using a fructose/oleic acid molar ratio of 1:5 [20], and an enzymatic loading of 0.51 IU/mL of solvent (*p*-NPB activity). After 12 h stirring in a Marconi incubator equipped with 360°- stirring carrousel, the biocatalysts were separated by centrifugation and the solvent (*tert*-butyl alcohol) was evaporated in an oven at 70 °C for 24 h. The unconverted fructose was then solubilized in distilled water and its concentration was determined by liquid chromatography as described above. The synthesis of fructose oleate (Figure 6) was also performed using an enzymatic loading of 8.4 IU/mL of solvent at the same experimental conditions described above (except that in this case the *tert*-butyl alcohol contained 1%, *v*/*v* of water) in order to decrease the reaction time, and consequently to increase the ester productivity. In this case, the ester conversion was monitored at different time intervals up to 8 h of reaction.

#### 3.2.7. Operational Stability Tests

In this set of experiments, the experimental conditions were those previously defined in this study, i.e., 35 °C, fructose/oleic acid molar ratio of 1:5, enzyme concentration of 8.4 IU/mL, 5 mL of *tert*-butyl alcohol containing 1% (*v*/*v*) of water, time reaction of 30 min for each cycle. After each cycle of reaction, the biocatalyst was recovered and washed with *tert*-butyl alcohol to remove unreacted substrates and products from the biocatalyst microenvironment. The unconverted fructose was quantified by liquid chromatography, as described previously.

## 4. Conclusions 

The chemical modification of the silica surface with hydrophobic/hydrophilic moieties was an important strategy to prepare active and stable biocatalysts by immobilizing microbial lipases from *Thermomyces lanuginosus* and *Pseudomonas fluorescens*. The lipases immobilized at low initial protein loading exhibited hyperactivation and were highly stable in *tert*-butyl alcohol. These biocatalysts were successfully used in the synthesis of fructose oleate in an organic/aqueous medium (*tert*-butyl alcohol/water) yielding high ester conversions and increased productivities. The strategy used in this study, employing a mixture of *tert*-butyl alcohol and water instead of toxic organic solvents (e.g., DMSO, pirydine, hexane, and others), can be considered as excellent alternatives to ester synthesis in organic media, allowing high conversion, higher solubilization of sugar, and high productivity of ester. Thus, this strategy combines a gain in performance of the enzyme and also allowing the development of a process free of toxic solvents.

## Figures and Tables

**Table 1:** Catalytic properties of the biocatalysts prepared by immobilization of PFL and TLL on mono and heterofunctional silica particles.

**Protein Loading = 1 mg/g of Support**						
**Support**	**TLL**			**PFL**		
	**IY ^d^ (%)**	**RA ^e^ (%)**	**HA ^f^ (IU/g)**	**IY ^d^ (%)**	**RA ^e^ (%)**	**HA ^f^ (IU/g)**
OS ^a^	88.6 ± 1.0	110.4 ± 0.4	104.7 ± 0.3	92.0 ± 1.8	208.1 ± 4.1	20.3 ± 0.7
OSGlu ^a^	89.2 ± 1.6	36.9 ± 1.3	35.2 ± 1.1	87.5 ± 0.6	110.3 ± 5.7	9.8 ± 0.5
OSEpx ^b^	84.3 ± 0.1	58.6 ± 1.3	75.5 ± 0.8	80.4 ± 3.6	157.1 ± 7.0	13.4 ± 0.9
OSGlx ^c^	86.9 ± 0.4	50.5 ± 3.5	43.8 ± 2.6	90.0 ± 1.5	139.7 ± 2.4	13.3 ± 1.1
**Protein Loading = 10 mg/g of Support**						
**Support**	**TLL**			**PFL**		
	**IY ^d^ (%)**	**RA ^e^ (%)**	**HA ^f^ (IU/g)**	**IY ^d^ (%)**	**RA ^e^ (%)**	**HA ^f^ (IU/g)**
OS ^a^	96.9 ± 0.3	93.3 ± 0.1	169.1 ± 9.2	89.6 ± 0.5	55.2 ± 0.1	132.7 ± 2.1
OSGlu ^a^	94.9 ± 0.2	47.6 ± 0.1	84.6 ± 0.6	90.3 ± 1.1	39.8 ± 0.1	96.4 ± 0.6
OSEpx ^b^	96.7 ± 0.8	56.1 ± 0.1	101.5 ± 1.6	88.0 ± 0.9	5.3 ± 1.2	12.6 ± 0.2
OSGlx ^c^	95.5 ± 0.6	33.9 ± 0.2	60.7 ± 1.6	90.3 ± 1.4	6.0 ± 1.0	14.6 ± 0.2

^a^ 25 °C, pH 7.0 (10 mM sodium phosphate buffer) for 6 h. ^b^ 25 °C, pH 7.0 (10 mM sodium phosphate buffer), followed by incubating at pH 10.0 (100 mM sodium carbonate buffer) for 3 h—Remaining epoxy groups were blocked with 3 M glycine solution (25 °C for 1 h). ^c^ 25 °C, pH 7.0 (10 mM sodium phosphate buffer), followed by incubating at pH 10.0 (100 mM sodium carbonate buffer) for 3 h—Remaining free aldehydes and Schiff’s bases were reduced with sodium borohydride (1 mg/mL, 25 °C for 1 h). ^d^ Immobilization yield. ^e^ Recovered activity. ^f^ Apparent hydrolytic activity.

**Table 2:** Solubility of fructose in *tert*-butyl alcohol/water systems at 35 °C.

Water Content (% *v*/*v*)	Soluble Fructose Concentration	
	g/L	mM
0	9.5 ± 0.1	52.7 ± 0.6
1	12.7 ± 0.3	70.5 ± 1.7
5	37.2 ± 0.8	206.5 ± 4.4
10	42.2 ± 2.1	234.2 ± 11.6

**Table 3:** Productivities of fructose oleate at 35 °C by enzymatic synthesis in a *tert*-butyl alcohol/water system. The reactions were catalyzed by both immobilized lipases for 12 h of reaction using a fructose:oleic acid molar ratio of 1:5 and 0.51 IU/mL of solvent.

Biocatalysts	Productivity (g/L.h) as Function of Water Amount (% *v*/*v*) in the Reaction Medium			
	0%	1%	5%	10%
PFL-OSEpx	0.06 ± 0.01			
PFL-OSGlx	0.39 ± 0.03	0.88 ± 0.05	1.18 ± 0.17	1.24 ± 0.17
PFL-OSGlu	0.28 ± 0.04	0.95 ± 0.02	0.81 ± 0.04	1.17 ± 0.14
PFL-OS	0.34 ± 0.03	0.95 ± 0.02	0.74 ± 0.05	0.86 ± 0.22
TLL-OSEpx	0.14 ± 0.01			
TLL-OSGlx	0.18 ± 0.01	0.96 ± 0.05	0.52 ± 0.07	0.50 ± 0.07
TLL-OSGlu	0.20 ± 0.01	0.92 ± 0.07	0.88 ± 0.02	1.06 ± 0.06
TLL-OS	0.32 ± 0.01	0.92 ± 0.04	0.94 ± 0.10	0.97 ± 0.06
